# Concomitant dual-site tDCS and dark chocolate improve cognitive and endurance performance following cognitive effort under hypoxia: a randomized controlled trial

**DOI:** 10.1038/s41598-023-43568-y

**Published:** 2023-09-30

**Authors:** Parisa Banaei, Vahid Tadibi, Ehsan Amiri, Daniel Gomes da Silva Machado

**Affiliations:** 1 Exercise Metabolism and Performance Lab (EMPL), Department of Exercise Physiology, Faculty of Sport Sciences, Razi University, University Avenue, Taq-e Bostan, Kermanshah, 6714414971 Iran; 2 Research Group in Neuroscience of Human Movement (NeuroMove), Department of Physical Education, Federal University of Rio Grande do Norte, Natal, RN 59078-970 Brazil

**Keywords:** Physiology, Environmental sciences, Medical research

## Abstract

Ten male cyclists were randomized into four experimental conditions in this randomized, cross-over, double-blind, and sham-controlled study to test the combined effect of acute dark chocolate (DC) ingestion and anodal concurrent dual-site transcranial direct current stimulation (a-tDCS) targeting M1 and left DLPFC on cognitive and whole-body endurance performance in hypoxia after performing a cognitive task. Two hours before the sessions, chocolate was consumed. After arriving at the lab, participants completed an incongruent Stroop task for 30 min in hypoxia (O_2_ = 13%) to induce mental fatigue, followed by 20 min of tDCS (2 mA) in hypoxia. Then, in hypoxia, they performed a time-to-exhaustion task (TTE) while measuring physiological and psychophysiological responses. Cognitive performance was measured at baseline, after the Stroop task, and during and after TTE. TTE in ‘DC + a-tDCS’ was significantly longer than in ‘white chocolate (WC) + a-tDCS’ and WC + sham-tDCS’. The vastus medialis muscle electromyography amplitude was significantly higher in ‘DC + a-tDCS’ and ‘DC + sham-tDCS’ than in ‘WC + sh-tDCS’. During and after the TTE, choice reaction time was significantly lower in ‘DC + a-tDCS’ compared to ‘WC + sh-tDCS’. Other physiological or psychophysiological variables showed no significant differences. The concurrent use of acute DC consumption and dual-site a-tDCS might improve cognitive and endurance performance in hypoxia.

By increasing altitude, the partial pressure of O_2_ decreases, which would limit oxygen delivery to the exercising muscles^1^ and the brain^2^. Numerous studies have shown that exercise performance is impaired in hypoxic conditions^3–7^. This hypoxia-induced decrement in exercise performance has been attributed to decreased arterial oxygen saturation (SpO_2_), increased pulmonary ventilation, and also increased afferent feedback from the working muscles which leads to the diminution of motor output from the central nervous system to the periphery^6–8^. In addition, hypoxia-induced impairment in physiological, psychological, cognitive, and perceptual responses to exercise has been reported in most of the previous studies^2,7,9–11^. Interestingly, current studies propose that in both moderate and severe hypoxia, the brain plays a pivotal role in regulating endurance performance, probably via different mechanisms^2^. The terms like "brain-hypoxic effect" or "the hypoxia-sensitive central component of fatigue" have been used in this regard to accentuating that in severe hypoxia, central mechanisms are the major cause of fatigue and endurance performance deterioration^2^. It has also been shown that in moderate hypoxia, the central drive from the brain to the working muscles decreases as a result of the hypoxia-induced increase in the discharge of inhibitory muscle afferents leading to a decrement in exercise performance^6^. The Psychobiological Model of fatigue during endurance exercise provides more support for this claim by assuming that rating of perceived exertion (RPE) and potential motivation, both processed in the brain, are important determinants of endurance performance^12^. In this regard, it has been reported that the levels of arousal and affective responses also play an important role in exercise performance as they could negatively impact potential motivation and RPE^13–15^ which are important psychophysiological markers for endurance exercise performance^16^. In fact, it has been demonstrated that the rate of decline in the affective responses is strongly correlated with the time to exhaustion (TTE) in the endurance cycling test^16^.

Studies have also demonstrated that hypoxia has a detrimental effect on cognitive function (i.e., impaired attentional ability, executive function, and memory function) and the psychophysiological homeostasis of the body^17,18^. Oxygen delivery to the brain through the neurovascular coupling (NVC) mechanism is one of the most important determinant factors of cognitive functions. It has been shown that the NVC might be negatively affected by hypoxia, culminating in reduced cognitive function^19^. It has also been indicated that the perturbation in psychophysiological homeostasis caused by hypoxic exposure results in mental fatigue^20^, which is defined as a psychobiological state characterized by feelings of tiredness/lack of energy at rest and/or increased RPE during subsequent whole-body endurance exercise^21,22^. Mental fatigue has been experimentally induced by performing a highly demanding cognitive task (e.g., Stroop test) for prolonged periods, in general ranging from 30 to 90 min^23^. In this context, a systematic review by Van Cutsem et al.^23^ found that mental fatigue decreased endurance performance (i.e., TTE and self-selected power output), which was associated with an increased RPE. Interestingly, they found no alterations in physiological responses associated with endurance performance [i.e., heart rate (HR), blood lactate, oxygen uptake, cardiac output, maximal aerobic capacity]^23^. In general, it could be concluded that mental fatigue negatively impacts physical and mental functions^21^. Accordingly, applying potentially effective strategies to counteract the detrimental effects of hypoxia and mental fatigue on various aspects of exercise performance has become a growing area of interest^7,17,20^.

Nutritional interventions are the most common strategies to counteract hypoxia-induced impairments in exercise performance^24^. In this regard, polyphenols are a nutritional component that has been proposed to have antioxidant, anti-inflammatory, and metabolic properties, and their ingestions might be beneficial for cognitive performance, brain functions, brain plasticity, and neuroprotection^25,26^. Alternatively, polyphenols can also have vasoactive effects likely due to their capacity to enhance the bioavailability and bioactivity of nitric oxide (NO), which could present interesting effects on increasing blood flow to the muscles and active brain areas during exercise that could improve exercise performance^27,28^. Cocoa is an important source of dietary polyphenols because its seeds contain high levels of polyphenols, which are bioactive compounds, besides fatty acids, vitamins, minerals, fiber, and several methylxanthine alkaloids^29,30^. Dark chocolate (DC) contains high amounts of cocoa, which makes it easier for daily consumption^31^. A recent review by Martín et al.^26^ has found that acute ingestion of DC improves several domains of cognitive performance in young adults, which are accompanied by increased cerebral blood flow and oxygenation. The efficacy of consuming DC in exercise performance, cognitive function, and reduction of fatigue in hypoxic conditions has also been reported^32,33^. Shaw et al.^33^ demonstrated that the consumption of DC caused a decrease in lactate levels and fatigue at the simulated altitude, with no change in endurance performance. Decroix et al.^32^ showed that consuming cocoa flavanols had a beneficial effect on prefrontal oxygenation during moderate-intensity exercise in both normoxia and hypoxia. In addition, several studies have reported that polyphenolic compounds can have a positive effect on cognitive function, especially executive function, attention, working memory, and processing speed^34–38^. However, the effect of acute DC ingestion on cognitive and endurance performance after performing a prolonged cognitive effort in hypoxia remains to be tested.

Moreover, anodal transcranial direct current stimulation (a-tDCS) has recently been suggested to boost exercise performance^39^ and cognitive function^40–42^. tDCS is a non-invasive, relatively inexpensive, portable, and easy to use, brain-modulating technique that involves applying a weak electrical current (up to 4 mA) to the scalp over a brain area of interest that can modulate spontaneous neuronal activity and excitability, in general, in a polarity-dependent manner^39,43^. The primary motor cortex (M1) and dorsolateral prefrontal cortex (DLPFC) are the most common areas investigated in previous studies mostly because of their substantial role in regulating exercise endurance performance and cognitive function^39,44^. Indeed, various studies have shown that stimulating either M1 or DLPFC improves various aspects of exercise performance^39,45–49^. The underlying mechanisms in this context have been reported to be increasing corticospinal excitability and neural drive to the periphery^50^, improving blood flow to the brain^51^, reducing RPE^49^, improving the psychophysiological responses^39^, and optimizing the information processing in the brain^52^. However, the effect of tDCS may depend on stimulation parameters (e.g., electrode montage, size, number, current intensity, and density), and previous studies using tDCS to boost physical performance used electrode montages either targeting M1 or DLPFC separately^43,46,53^. In this context, the anodal unihemispheric concurrent dual-site tDCS (a-tDCS_UHCDS_) was recently proposed as an effective strategy for simultaneously stimulating M1 and DLPFC yielding a greater increase in corticospinal excitability compared to the isolated stimulation of M1 and DLPFC^54,55^. It is noteworthy that this increase in corticospinal excitability lasted for 24 h after a-tDCS_UHCDS_^54^. Talimkhani et al.^56^ provided further support for the effectiveness of a-tDCS_UHCDS_ by showing its positive effect on the acquisition of cognitive skills and functions in healthy subjects. However, from a practical point of view, if this greater corticospinal excitability translates into improved performance in whole-body endurance exercise (specifically under hypoxic conditions) also remains to be tested.

Thus, considering the detrimental effect of both hypoxia and mental fatigue on cognitive and physical performance, and the overlapping between its underlying mechanisms with the action mechanisms of DC and tDCS, it is possible to speculate that the combination of DC and a-tDCS_UHCDS_ would have a synergistic effect to counteract the detrimental effects of hypoxia and mental fatigue on cognitive and exercise performance. Therefore, we aimed to analyze the acute effect of combined DC supplementation and a-tDCS_UHCDS_ on cognitive performance, whole-body endurance exercise performance, physiological, and psychophysiological responses under hypoxia after performing a mentally demanding task.

## Results

### tDCS-induced sensations and blinding

All 10 participants received the experimental conditions according to the randomization. The participants reported no serious side or adverse effects. The most common sensations reported were itching and burning. No other sensation beyond the ones listed in the questionnaire was reported. All participants reported that these sensations were located on the head, starting and ending at the beginning of the stimulation (Table 1). There was no significant difference in sensations intensity, beginning and end of the sensations, and perceived effect on performance among conditions. All participants reported these sensations positively affected their performance ranging from slightly to considerably. The percentage of correct guesses regarding the tDCS condition differed among conditions (χ^2^^2^ = 30.0; *p* < 0.001), with the active tDCS conditions (DC + a-tDCS = 100% and WC + a-tDCS = 100%) significantly higher than sham conditions (DC + sh-tDCS = 0% and WC + sh-tDCS = 0%; all *p*s = 0.002). This was because all individuals (100%) thought they had been stimulated in all four conditions. Hence, considering the similar tDCS-induced sensations and active guess rate, it can be assumed that the study blinding protocol was effective.

**Table 1:** tDCS-induced sensations and the general sensation index (discomfort) felt by participants (n = 10).

Sensation	DC + a-tDCS			WC + a-tDCS			DC + sh-tDCS			WC + sh-tDCS			*χ2*	*p*
	M ± SD	Median (IQR)	*n* (%)	M ± SD	Median (IQR)	*n* (%)	M ± SD	Median (IQR)	*n* (%)	M ± SD	Median (IQR)	*n* (%)		
Itchiness	1.0 ± 0.5	1.0 (1.0–1.0)	9 (90)	1.2 ± 0.4	1.0 (1.0–1.3)	10 (100)	1.1 ± 0.3	1.0 (1.0–1.0)	10 (100)	1.2 ± 0.4	1.0 (1.0–1.3)	10 (100)	3.7	.30
Pain	0.0 ± 0.0	0.0 (0.0–0.0)	0 (0)	0.0 ± 0.0	0.0 (0.0–0.0)	0 (0)	0.0 ± 0.0	0.0 (0.0–0.0)	0 (0)	0.0 ± 0.0	0.0 (0.0–0.0)	0 (0)	N/A	N/A
Burning	0.7 ± 0.5	1.0 (0.0–1.0)	7 (70)	0.3 ± 0.5	0.0 (0.0–1.0)	3 (30)	0.3 ± 0.5	0.0 (0.0–1.0)	3 (30)	0.6 ± 0.7	0.5 (0.0–1.0)	5 (50)	5.8	.12
Warmth/Heat	0.0 ± 0.0	0.0 (0.0–0.0)	0 (0)	0.0 ± 0.0	0.0 (0.0–0.0)	0 (0)	0.1 ± 0.3	0.0 (0.0–0.0)	1 (10)	0.0 ± 0.0	0.0 (0.0–0.0)	0 (0)	3.0	.39
Pinching	0.0 ± 0.0	0.0 (0.0–0.0)	0 (0)	0.0 ± 0.0	0.0 (0.0–0.0)	0 (0)	0.0 ± 0.0	0.0 (0.0–0.0)	0 (0)	0.0 ± 0.0	0.0 (0.0–0.0)	0 (0)	N/A	N/A
Iron taste	0.0 ± 0.0	0.0 (0.0–0.0)	0 (0)	0.0 ± 0.0	0.0 (0.0–0.0)	0 (0)	0.0 ± 0.0	0.0 (0.0–0.0)	0 (0)	0.0 ± 0.0	0.0 (0.0–0.0)	0 (0)	N/A	N/A
Fatigue	0.0 ± 0.0	0.0 (0.0–0.0)	0 (0)	0.0 ± 0.0	0.0 (0.0–0.0)	0 (0)	0.0 ± 0.0	0.0 (0.0–0.0)	0 (0)	0.0 ± 0.0	0.0 (0.0–0.0)	0 (0)	N/A	N/A
Other	0.0 ± 0.0	0.0 (0.0–0.0)	0 (0)	0.0 ± 0.0	0.0 (0.0–0.0)	0 (0)	0.0 ± 0.0	0.0 (0.0–0.0)	0 (0)	0.0 ± 0.0	0.0 (0.0–0.0)	0 (0)	N/A	N/A
Discomfort	1.7 ± 0.8	2.0 (1.0–2.0)	–	1.5 ± 0.7	1.0 (1.0–2.0)	–	1.5 ± 0.7	1.0 (1.0–2.0)	–	1.8 ± 0.8	2.0 (1.0–2.3)	–	3.2	.36
Start	1.0 ± 0.0	1.0 (1.0–1.0)	–	1.2 ± 0.4	1.0 (1.0–1.3)	–	1.2 ± 0.4	1.0 (1.0–1.3)	–	1.2 ± 0.4	1.0 (1.0–1.3)	–	2.3	.52
End	1.2 ± 0.4	1.0 (1.0–1.3)	–	1.3 ± 0.5	1.0 (1.0–2.0)	–	1.3 ± 0.5	1.0 (1.0–2.0)	–	1.3 ± 0.5	1.0 (1.0–2.0)	–	0.4	.95
Affect performance	2.7 ± 0.5	3.0 (2.0–3.0)	10 (100)	2.5 ± 0.5	2.5 (2.0–3.0)	10 (100)	2.5 ± 0.5	2.5 (2.0–3.0)	10 (100)	2.4 ± 0.5	2.0 (2.0–3.0)	10 (100)	4.4	.22

*M ± SD* mean and standard deviation; *IQR* interquartile range; *DC* dark chocolate; *WC* white chocolate; *a-tDCS* anodal transcranial direct current stimulation; *sh-tDCS* sham transcranial direct current stimulation; *n(%)* indicates the number and percentage of participants who experienced a particular sensation.

The mean value of the study variables under four experimental conditions is presented in Table 2.

**Table 2:** Comparison in performance, physiological and psychophysiological variables in all four experimental conditions (*n* = 10).

Variables	Experimental Conditions			
	DC + a-tDCS	WC + a-tDCS	DC + sham-DCS	WC + sh-tDCS
Time to exhaustion (Min: Sec)	20:38 ± 9:48*^#^	14:02 ± 7:00	19:22 ± 11:02	13:38 ± 6:31
Heart rate (Beats Per Min)	164 ± 33.2	158.8 ± 30.2	166.2 ± 24.2	158.2 ± 31
SpO_2_ (%)	77.65 ± 5.8	75.35 ± 5.6	78.62 ± 6.1	77.23 ± 6.3
RPE (0–100, Borg Scale)	56.5 ± 18.59	63.13 ± 18.9	63.52 ± 21.2	73.76 ± 18.5
EMG of VL (% of MVC)	28.98 ± 5.4	30.76 ± 10.7	26.68 ± 15.1	26.83 ± 12.9
EMG of VM (% of MVC)	39.77 ± 12.5*	35.48 ± 15.2	47.4 ± 23.7*	24.2 ± 7.03
EMG of RF (% of MVC)	13.73 ± 8.7	15.5 ± 7.4	15.43 ± 11.4	13.68 ± 7.3
CRT-baseline (Milliseconds)	354 ± 63.1	364.3 ± 80.9	361.4 ± 94.5	369.1 ± 89.8
CRT-during (Milliseconds)	334 ± 53.5*	401 ± 74.9	402 ± 44.1	470 ± 59
CRT-after (Milliseconds)	302.6 ± 57.4*	325.6 ± 74.3	310.1 ± 88.2	394 ± 29.5
FAS (1–6, Felt arousal scale)	2.75 ± 0.74	3.12 ± 1.1	3.17 ± 0.84	2.52 ± 1.08
Affective responses ( − 5 to +5)	−1.42 ± 2.1	−1.52 ± 1.05	−0.64 ± 1.6	−1.22 ± 2

Data presented as mean ± standard deviation

*tDCS* Transcranial direct current stimulation; *DC* Dark chocolate; *WC* White chocolate; *SpO*_*2*_* RPE* Rating of perceived exertion; *EMG* Electromyography; *CRT* Choice reaction time; *FA* Felt arousal.

*Significant difference compared to ‘WC + sh-tDCS’.

^#^Significant difference compared to ‘WC + a-tDCS’.

### Effect of the demanding cognitive task

**Figure 1:**
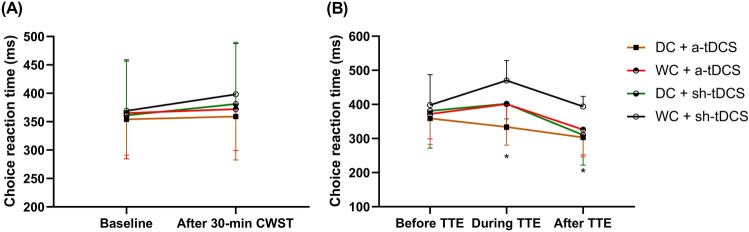
Reaction time changes in the four experimental conditions. Comparison of choice reaction time performance (**A**) at baseline in normoxia and after performing a modified Stroop task (incongruent only) for 30 min in hypoxia among experimental conditions. Choice reaction time performance (**B**) before, during, and immediately after the time to exhaustion test (TTE) under hypoxia (O_2_ = 13%) after performing an incongruent-only Stroop task for 30 min and receiving anodal or sham Transcranial Direct Current Stimulation for 20 min in hypoxia. * = “DC + a-tDCS" significantly higher than “WC + sh-tDCS” (*p* = 0.003). *DC* dark chocolate; *WC* white chocolate; *a-tDCS* anodal transcranial direct current stimulation; *sh-tDCS* sham transcranial direct current stimulation.

Performing the incongruent only Stroop task for 30 min did not induce a state of mental fatigue as we found no significant main effect of time *(F*_*(1,72)*_ = *0.635; p *_=_ *0.428)*, condition *(F*_*(3,72)*__=_ *0.335; p *_=_ *0.8)*, or time x condition interaction *(F*_*(3,72)*__=_ *0.088; p *_=_ *0.967)* on CRT (Fig. 1A).

### Effect of DC and a-tDCS_UHCDS_ on cognitive performance

Our results showed a significant condition x time interaction *(F*_*(6,54)*_= *2.39, p*_=_*0.04, ɳ*^*2*^_*p*_= *0.210)* on CRT, but no main effect of either time or condition separately. Hence, we explored this result to analyze the main effect of the condition at each time point. The results demonstrated that there was no significant difference in CRT at baseline among experimental conditions (*F*_*(3,27)*_= *0.112, p *= *0.95, ɳ*^*2*^_*p*_=* 0.012; *Fig. 1A). Inversely, there was a significant effect of condition on CRT during TTE in hypoxia (*F*_*(3,27)*_= *9.42, p *= *0.0001, ɳ*^*2*^_*p*_= *0.512; *Fig. 1B), which was significantly lower in ‘DC + a-tDCS’ compared to ‘WC + sh-tDCS’ condition (*p *= *0.008, d*_*av*_= *2.4, Δ *= *− 28.93%*) with no significant differences among other conditions (*p ˃ 0.05*). There was also a significant effect of condition on CRT after exhaustion in hypoxia (*F*_*(3,27)*_= *4.61, p *= *0.01, ɳ*^*2*^_*p*_= *0.339; *Fig. 1B), which was significantly lower in ‘DC + a-tDCS’ compared to ‘WC + sh-tDCS’ condition (*p *_=_ *0.016, d*_*av*_= *1.04, Δ*_= _*− 23.19%)* with no significant among other conditions (*p ˃ 0.05*).

### Effect of DC and a-tDCS_UHCDS_ on endurance performance

**Figure 2:**
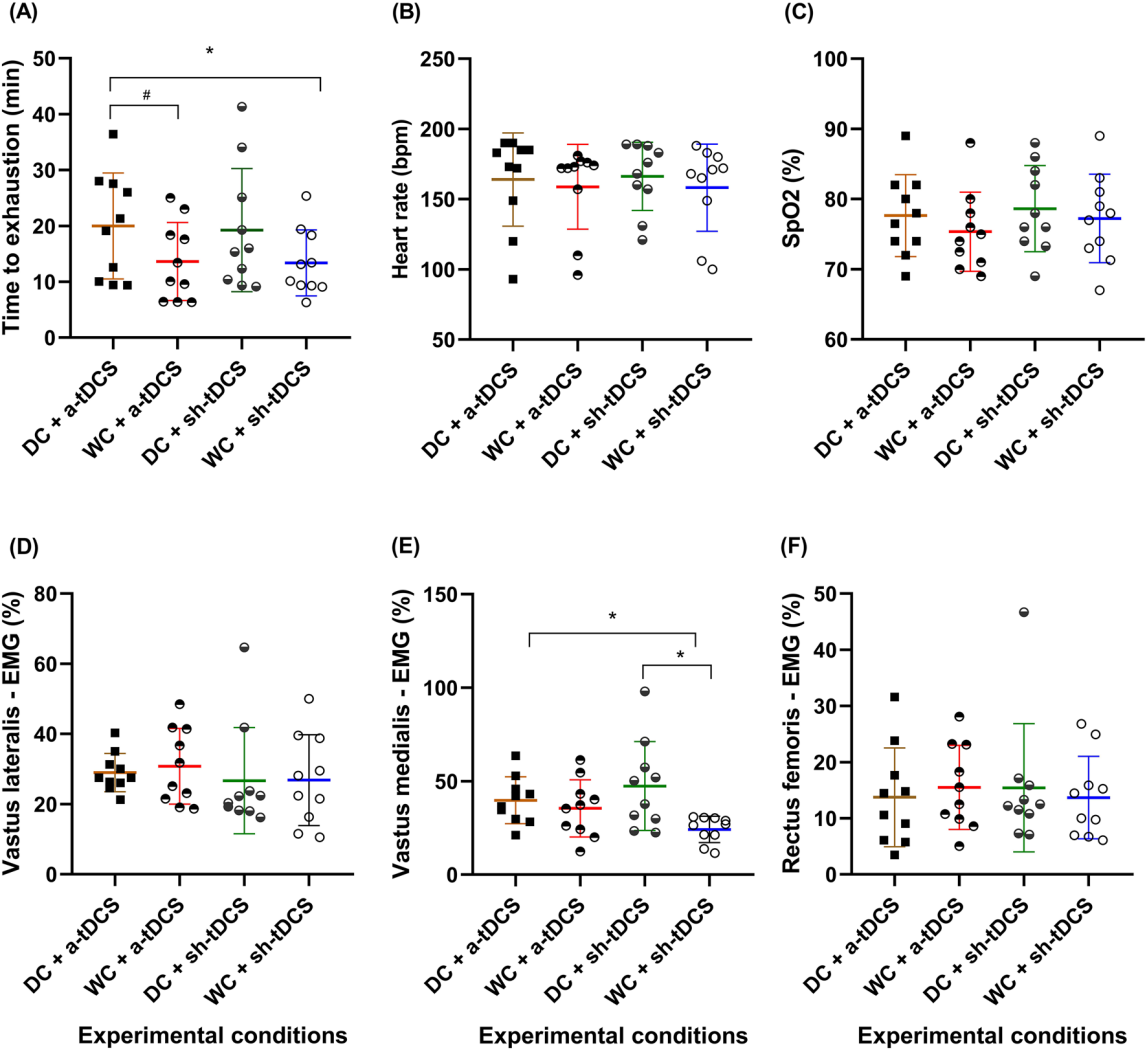
Endurance performance and physiological responses in the four experimental conditions. Time to exhaustion test (**A**), heart rate (**B**), blood oxygen saturation (SpO_2_; **C**), and electromyographic (EMG) amplitude of the vastus lateralis (**D**), vastus medialis (**E**), and rectus femoris (**F**) during the endurance cycling task under four experimental conditions in hypoxia. * = significantly different from WC + sh-tDCS (*p* < 0.05); ^#^ = significantly different from WC + a-tDCS (*p* < 0.05). *DC* dark chocolate; *WC* white chocolate; *a-tDCS* anodal transcranial direct current stimulation; *sh-tDCS* sham transcranial direct current stimulation.

The concomitant use of DC and a-tDCS_UHCDS_ improved endurance performance in hypoxia, as there was a significant effect of the conditions (*F*_*(3,27)*=_*7.34, p*_=_*0.001, ɳ*^*2*^_*p*=_*0.449; *Fig. 2A), with a significantly higher TTE in ‘DC + a-tDCS’ than in ‘WC + a-tDCS’ and ‘WC + sh-tDCS’ conditions *(p*_=_*0.002, d*_*av*=_*0.77, Δ*_=_*46.7%; p*_=_*0.038, d*_*av*=_*0.85, Δ*_=_*49.3%, respectively)*. No significant differences were observed among other conditions *(p˃0.05).*

### Effect of DC and a-tDCS_UHCDS_ on physiological responses

DC and/or a-tDCS_UHCDS_ increased EMG amplitude during TTE in hypoxia, as there was a significant effect of the condition on the EMG amplitude of VM muscle during TTE in hypoxia (*F*_*(3,27)*_= *4.7, p *= *0.009, ɳ*^*2*^_*p*_= *0.344; *Fig. 2E). A significantly higher VM EMG amplitude in ‘DC + a-tDCS’ (*p *= *0.049, d*_*av*_= *1.59, Δ *= *64.3%*) and ‘DC + sh-tDCS’ (*p *= *0.045, d*_*av*_= *1.5, Δ*_=_*95.8%*) compared to the ‘WC + sh-tDCS’ condition, with no other significant difference *(p˃0.05)*. However, there was no significant difference in the EMG amplitude of RF (*F*_*(1.46,13.14)*_=* 0.180, p *=* 0.7, ɳ*^*2*^_*p*_=* 0.02; *Fig. 2F) and VL muscles* (F*_*(3,27)*_=* 0.315, p *= *0.8, ɳ*^*2*^_*p*_= *0.034; *Fig. 2D). Similarly, there was no significant effect of the conditions on HR *(F*_*(3,27)*_= *0.141, p *= *0.93, ɳ*^*2*^_*p*_= *0.015; *Fig. 2B) and SpO_2_ *(F*_*(3,27)*_= *2.08, p *= *0.12, ɳ*^*2*^_*p*_= *0.188; *Fig. 2C*)* during TTE in hypoxia among conditions.

### Effect of DC and a-tDCS_UHCDS_ on psychophysiological responses

**Figure 3:**
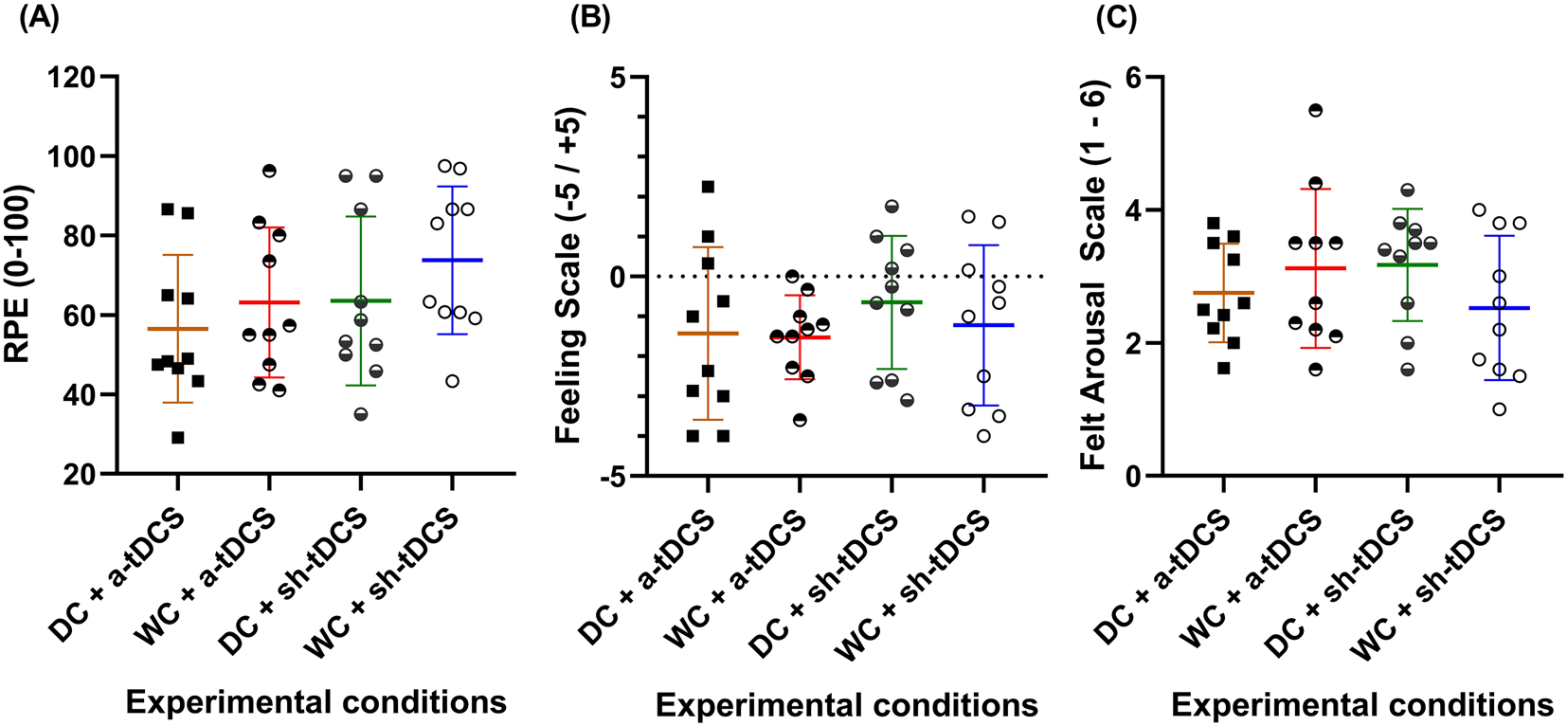
Psychophysiological responses to exercise in the four experimental conditions. Ratings of perceived exertion (RPE; **A**), affective responses (**B**), and felt arousal (**C**) during the endurance cycling task under four experimental conditions in hypoxia. DC dark chocolate; WC white chocolate; a-tDCS anodal transcranial direct current stimulation; sh-tDCS sham transcranial direct current stimulation.

DC and/or a-tDCS_UHCDS_ did not change psychophysiological responses during TTE in hypoxia, as there was no significant effect of condition on RPE *(F*_*(3,27)*_= *1.41, p *= *0.25, ɳ*^*2*^_*p*_= *0.114),* affective responses *(F*_*(3,27)*_= *0.64, p *= *0.59, ɳ*^*2*^_*p*_= *0.066),* or FAS *(F*_*(3,27)*_= *1.08, p *= *0.37, ɳ*^*2*^_*p*_= *0.108)* during TTE in hypoxia (Fig. 3A–C)*.*

## Discussion

The main results of the present study were that (1) ‘DC + a-tDCS’ resulted in longer TTE in hypoxia than ‘WC + a-tDCS’ and ‘WC + sh-tDCS’ conditions, (2) with greater EMG activity of the VM muscle, and (3) improved cognitive performance during and after TTE. These results suggest the possibility of a synergistic effect between DC and a-tDCS_UHCDS_. To the best of the authors’ knowledge, this is the first study to test the combined effect of DC and tDCS on cognitive and endurance performance in hypoxia after performing a prolonged cognitive effort, and its related physiological and psychophysiological responses.

First, it is important to note that despite performing the modified incongruent Stroop task for 30 min in hypoxia, cognitive performance was unchanged over time *(*Fig. 1A*)*. This indicates that a state of mental fatigue was not induced in the present study. One could argue that this result was due to chocolate consumption 2 h before the experimental sessions, but no difference was found between DC and WC (‘placebo’) conditions. It is noteworthy that various studies succeed in inducing mental fatigue using the same cognitive task and duration^57–59^, including in professional road cyclists^60^. Hence, despite the potential negative effect of hypoxia on cognitive performance^61,62^, which would have exacerbated the induction of mental fatigue in the current study^63,64^, it did not occur. Therefore, we did not assume that mental fatigue played a role in this study, and we used the term "prior cognitive effort" to refer to the attempt to induce mental fatigue. When attempting to induce mental fatigue in hypoxia in endurance-trained athletes, future studies should use longer durations of cognitive effort.

On the other hand, DC + a-tDCS improved cognitive performance during and after TTE in hypoxia. This is partially in line with previous studies showing cognitive improvements at rest with either DC^26,34,65^ or tDCS isolated^40–42^. Talimkhani et al.^56^ also showed that a-tDCS decreased the reaction time. However, different from previous studies we combined a-tDCS and DC and also assessed cognitive performance during and after an exhaustive endurance task, which is a novel contribution of the current study. This result is particularly important since cognitive performance has been deemed paramount for endurance exercise regulation and a determinant for the occurrence of exercise-induced fatigue^23,57^. Interestingly, the improved cognitive performance with DC + a-tDCS was accompanied by improved exercise performance (i.e., longer TTE duration) and, despite sustaining exercise for longer, cognitive performance was still preserved during and immediately after exhaustion. Hence, one possible explanation for improved endurance performance could be due to a preserved and/or improved cognitive performance that would be associated with greater top-down control of exercise performance.

The improved cognitive performance with DC + a-tDCS might be due to increased blood flow and oxygenation to the brain^26^. The consumption of cocoa flavonols, which are present in DC, a subgroup of polyphenols with antioxidant capacity, may cause NO-dependent vasodilation^66^. The regulation of NO metabolism through nutritional interventions can improve sports performance and reduce fatigue in both normoxia and hypoxia conditions^66,67^. Indeed, Maschelin et al.^68^ showed improvement in SpO_2_ and TTE after maximal exercise tests in hypoxic conditions (O_2_ = 11%) with 6-day consumption of nutritional supplements aiming at increasing plasma NO. However, we measured neither brain blood flow nor oxygenation or variables related to NO and, thus, this possible mechanism is speculative.

We found improved endurance performance (i.e., longer TTE) in hypoxia with DC + a-tDCS. This is also a novel finding of the present study. It should be noted that only DC + a-tDCS improved cognitive and endurance performance. Thus, we propose that this could be a synergistic effect since neither DC alone (DC + sh-tDCS) nor tDCS alone (WC + a-tDCS) improved cognitive or endurance performance. Despite previous studies have shown that a-tDCS targeting M1 or DLPFC separately improved endurance performance in cycling^39,69^, other studies found no change in endurance performance targeting the same brain regions^43,70^, which aligns with our finding that tDCS alone did not improved endurance performance. It is noteworthy that all previous studies have evaluated the effect of tDCS on exercise performance under normal ambient conditions and only a single recent exception assessed the effect of single-site tDCS on exercise performance in hypoxia and found improved endurance performance with DLPFC but not M1^71^. This is particularly important given that hypoxia provides important environmental changes that differ substantially from normoxia conditions. Considering DC, no study has shown so far that acute ingestion of DC improves endurance performance either in normoxia or in hypoxia^72^, which is also in line with our findings that DC alone did not improve endurance performance.

One possible mechanism for this synergistic effect is that the possible vascular effect of DC supplementation (i.e., vasodilation) allowed for the neuromodulatory effect of a-tDCS to take place. Theoretically, a tDCS-induced increase in corticospinal excitability and the change in the use of motor units can lead to an improvement in TTE^73^. Interestingly, a-tDCS_UHCDS_ was shown to induce greater corticospinal excitability compared to the isolated stimulation of M1 and DLPFC^54^, which lasted for 24 h after a-tDCS. However, it seems to have only taken place when associated with DC in the current study. Some studies suggest that the mechanisms associated with longer exercise tolerance mediated by a-tDCS stimulation are increased intracortical facilitation and M1 excitability^74–76^. Hence, the form of tDCS used in the present study targeting to stimulate simultaneously M1 and DLPFC seems to be a promising montage for endurance performance enhancement.

We found an increased EMG of the VM muscle under both DC + a-tDCS and DC + sh-tDCS compared to WC + sh-tDCS, but no difference in EMG of the RF and VL. Few studies analyzed the effect of tDCS on EMG during an endurance task, which limits comparisons. Vitor-Costa et al.^45^ found no change in EMG of the VL and RF during a TTE test after a-tDCS targeting M1 despite improved endurance performance. It should be noted that the EMG of VM muscle was not measured in that study, which limits comparison^45^. Previous studies showed that the greatest activity for the VL and VM occurs during the propulsion phase in cycling (knee extension)^77^, which is the most important phase of the cycling cycle. EMG of a given muscle represents motor units’ recruitment and/or rate of discharge of the active motor units, thus, being a surrogate measure of the descending neural drive from motor areas of the brain, especially M1. Hence, we could speculate that both DC + a-tDCS and DC + sh-tDCS increased the neural drive to the quadriceps muscle, especially the VM, during the propulsion phase. The same mechanisms explained above might be involved in these results, however, our outcome measures do not allow us to firmly establish that.

On other hand, there was no change in HR, SpO_2_, and RPE in the present study. These results align with previous studies with acute or sub-acute DC supplementation that found no changes in the mentioned physiological parameters^78–80^. Studies with tDCS have shown contrasting results, with some studies finding significantly lower HR and RPE at lower intensities or at intermediate time points^49,69^, while others found no changes^43,70^. Considering that studies with tDCS were performed in normoxia, SpO_2_ was not measured. The difference between studies might be related, for instance, to the participant’s characteristics such as training and/or fitness status and/or tDCS parameter used. In addition, considering that HR and RPE were analyzed considering the mean of the entire test duration, we suggest that a TTE lasting ~ 45% longer in DC + a-tDCS condition with similar HR and RPE should be considered a result of modulation in these variables as well. For instance, despite we did not perform this analysis, if we consider HR and RPE relative to each TTE duration the values in DC + a-tDCS condition would be probably lower than the other conditions^81,82^. Finally, the fact that SpO_2_ was not changed by any intervention could be explained by the type of measure performed. Considering that blood flow is precisely regulated to either increase or decrease according to the tissue needs, the fingertip pulse oximeter is not able to detect changes that would occur in the active muscles or the brain. It could only measure systemic changes or the fingertip level. Other measures of muscle and brain blood flow and oxygenation are suggested for future investigations. Finally, we found no difference in the affective response (FS) or perceived activation (FAS) during the TTE test. To the best of the authors' knowledge, despite the rate of decline in the affective responses being strongly correlated with TTE in cycling^16^, no study assessed the effect of DC or tDCS on these variables, which limits the comparisons. A similar rationale used to RPE (i.e., perceptual response relative to TTE duration) could explain the FS and FAS responses.

The interpretation and generalization of the findings of the present study should be made with caution since the participants were endurance-trained male cyclists. In addition, the lack of a measure of brain activity (e.g., EEG, fNIRS), corticospinal excitability, muscle, and brain blood flow, and oxygenation does not allow us to fully understand the mechanisms involved in the present findings. Future studies should consider these limitations and also expand the present findings, for instance, to higher levels of hypoxia. On the other hand, the strengths of the present study include a large array of outcome variables assessed and a complex experimental design testing the isolated and combined effects of DC and tDCS on cognitive and endurance performance in hypoxia and its related physiological and psychophysiological responses in professional athletes. From a practical perspective, if consistently replicated, the present findings indicate that athletes and coaches can use DC supplementation and tDCS to boost endurance performance in hypoxia.

The present study showed a possible acute synergistic effect of a-tDCS targeting concurrently M1 and left DLPFC along with the consumption of dark chocolate on improving whole-body endurance performance after prior cognitive effort, with greater EMG of VM muscle, cognitive performance during and after endurance TTE under hypoxia, despite unchanged HR, SpO_2_, and RPE, affective responses, and arousal in professional male cyclists. Therefore, these two types of interventions can be used in conditions of hypoxia and mental fatigue to improve cognitive and physiological factors in trained individuals.

## Methods

### Participants

Ten endurance-trained male cyclists voluntarily participated in this randomized, sham-controlled, and double-blind trial. The general characteristics of the participants are presented in Table 3. Participants were recruited from the cycling clubs of the city of residence, all of which participated in regular training at a high level and were involved in most of the competitions at regional and national levels for ≥ 3 years, with most being sponsored. The sample size was calculated using G*Power (Version 3.1.9.2, Kiel, Germany) for a repeated-measure ANOVA within factors as follows: α = 0.05; power (1-β err prob): 0.80; effect size f: 0.4; the number of groups = 1; correlation among repeated measures = 0.5, and non-sphericity correction = 1. The expected effect size for calculating the sample size was based on the meta-analysis by Machado et al.^39^. Accordingly, a sample of 10 participants was required. Considering the dropout rate of 20%, 12 participants were recruited for the present study, but 2 participants withdrew from the study for personal reasons. The inclusion criteria were as follows: professional male cyclists, no stay in altitude > 2000 m and no blood donation in the previous two months, no history of neurological diseases, no implanted medical devices or pacemakers in the body, no cardiovascular or orthopedic disease, no use of tobacco, drugs, and alcohol. The exclusion criteria were not completing the study protocol, injuries related or not to the study that could influence the outcome variables, and consuming dark chocolate, cocoa, and foods containing flavones on the days of the experiment. All the participants gave written informed consent to the study. The study was approved by the Institutional Ethics Committee and was conducted following the declaration of Helsinki. The study was approved by the Ethics Committee of Razi University Kermanshah, Iran (IR.RAZI.REC.1401.005) and registered in the Iranian Registry of Clinical Trials (IRCT id: IRCT20220417054556N1, Registration Date: 27/07/2022). The first participant was included on 07/08/2022, and the trial was terminated on 28/09/2022.

**Table 3:** General characteristics of the participants (n = 10).

Variables	Mean ± SD
Age (years)	23.5 ± 1.13
Body mass (kg)	78.04 ± 13.94
Height (cm)	181.4 ± 2.6
Body mass index (kg/m^2^)	23.64 ± 2.8
Body fat (%)	17.48 ± 5.7
Fat mass (kg)	13.67 ± 6.3
Fat-free mass (kg)	64.37 ± 10.2
Peak power output (W, in hypoxia)	311.35 ± 33.4

### General experimental procedure

**Figure 4:**
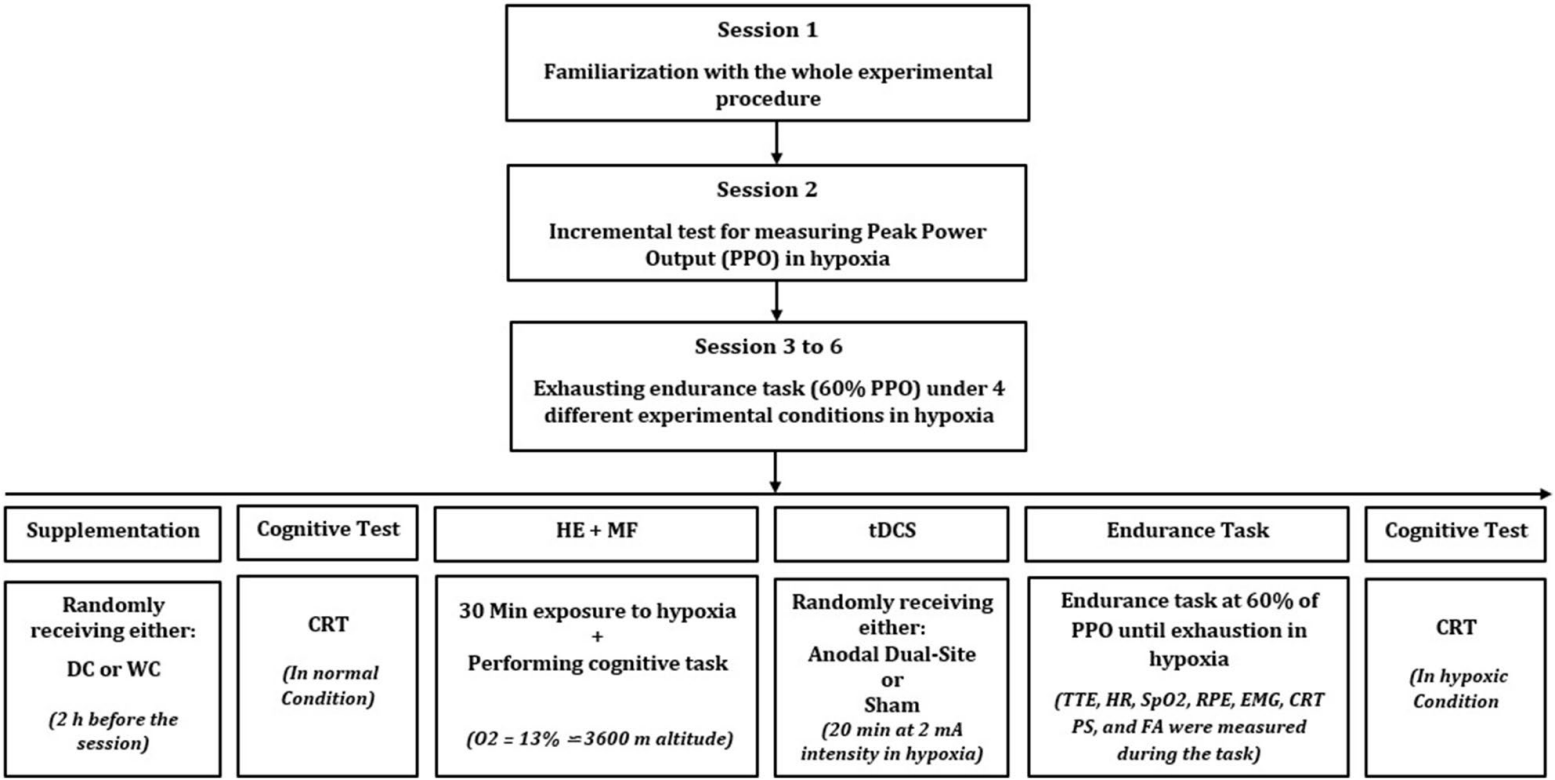
Study flowchart. *PPO* Peak Power Output; *CRT* Choice Reaction Time; *tDCS* Transcranial Direct Current Stimulation; *TTE* Time to Exhaustion; *HR* Heart Rate; *SpO*_*2*_ Blood Oxygen Saturation; *RPE* Rating of Perceived Exertion; *EMG* Electromyography; *PS* Pleasure Sensation (i.e., affective responses); *FA* Felt Arousal.

The participants visited the laboratory on six different occasions at one-week intervals. In the first session, participants underwent an anthropometric assessment and were familiarized with the whole experimental procedure. An individual not involved in the research team randomized the order of four experimental conditions using the Latin Square method. The participants and research team were blinded to the order of the experimental conditions throughout the study. In the second session, peak power output (PPO) was measured using an incremental cycling test in hypoxia (O_2_ = 13%). From the 3rd to 6th visits, the participants performed a time to exhaustion (TTE) cycling test with 60% of PPO in hypoxia under four different experimental conditions in a randomized order: (1) DC + anodal-tDCS, (2) white chocolate (WC) + anodal-tDCS, (3) DC + sham-tDCS, and (4) WC + sham-tDCS. Accordingly, two hours before coming to the lab for each experimental session, participants ingested either DC or WC. Upon arrival at the lab, participants first performed the choice reaction time (CRT) test as a measure of cognitive performance. Subsequently, the maximal isometric voluntary contraction (MIVC) test of knee extensor muscles was performed for normalizing the EMG data of that experimental session. Participants were then exposed to a hypoxic condition (O_2_ = 13%) while performing a color-word Stroop test (CWST) to induce mental fatigue for 30 min, followed by the CRT test. Following that, tDCS was applied to target areas for 20 min while still in hypoxia. Accordingly, each participant was under hypoxia for 50 min before performing the TTE. After tDCS, participants performed the TTE at 60% of PPO until voluntary exhaustion under the hypoxic condition. During the task, HR and SpO_2_ were measured constantly and, RPE, CRT, affective responses (Feeling Scale; FS), and perceived activation (Felt Arousal Scale; FAS) were measured every 3 min. Electromyographic activity (EMG) of the rectus femoris (RF), vastus lateralis (VL), and vastus medialis (VM) muscles were measured during TTE. After exhaustion, participants reported RPE, FS, and FAS, and then performed the CRT test under hypoxia. The whole experimental procedure is depicted in Fig. 4.

### Maximal incremental test

In the second session, after 30 min being under hypoxia at rest, participants underwent the maximal incremental test (Astrand Test for Men) on a cycle ergometer (Cyclus 2, RBM Elektronik-Automation GmbH, Leipzig, Germany) to measure PPO under the hypoxic condition. The test was started with 100 watts and a cadence of 60 rpm for three minutes and 50 watts increments every three minutes^83^. The test was performed until volitional fatigue. During the test, participants reported their RPE. The test was considered maximum if two of the following criteria were achieved: (1) ≥ 90% of maximal predicted HR (220-age), (2) inability to maintain the pedal cadence of 60 rpm for more than 5 s (3) RPE ≥ 90. PPO was determined as follows: PPO = *W*_*out*_ + (t/180) * 50 [*W*_*out*_*:* workload of the last completed stage; *t:* time in the final stage in seconds]^45^.

### Dark chocolate supplementation

In the DC conditions (DC), 2 h before the experimental sessions participants consumed ~ 467 kcal of a typical commercial 70% cocoa product (Nestlé Noir 70%) containing the ingredients cocoa liquor, sugar, cocoa butter, milk fat, lecithin and vanilla^84^. In the control conditions (WC), participants consumed ~ 487 kcal of WC closely matched to DC in terms of CHO, fat, and calories. Importantly, WC was used as a control for DC because it contains no cocoa butter or cocoa liquor^80^, which are the components that could potentially generate the effects. Previous studies using WC as a placebo, used the method of alkalizing cocoa for coloring^33,85^. However, due to the technical unavailability of an alkalizing method, participants were not blinded concerning the chocolate supplementation condition (e.g., they knew they were consuming either DC or WC). This same method was used in previous studies^80,85,86^. It should be noted that participants were not given any hint regarding the superiority of either DC or WC in order to prevent possible “expectation effect” bias. Also, participants did not receive any feedback on their performance for any outcome measure. Only after the end of the study participants were fully debriefed concerning the study hypothesis and their respective performance.

### Cognitive function—choice reaction time

The Visual Choice Reaction Time Apparatus (Model 63035A, Lafayette Instrument Company, Indiana, USA) with a four-choice compatible stimulus–response paradigm was used to measure cognitive performance. Three visual stimuli (lights turning on) were manually given to the participants, and they were instructed to respond as quickly as possible by pushing the corresponding button on the response panel. The reaction time in each stimulation was recorded and the mean value of 3 efforts was calculated. The CRT was performed at the beginning of each experimental session in normoxia, after the prolonged cognitive effort, and during and after TTE in hypoxia.

### MIVC

At the beginning of each session, participants performed 3–5 s knee extension MIVC three times with a 150-s rest in between on a custom-made chair with knee and hip fixed at 90° as recommended for VL, VM, and RF muscles MIVC test^87^. The best performance in MIVCs was recorded for normalizing the EMG signals of that session.

### EMG

The surface EMG signals were collected strictly according to the recommended standards^88,89^. In each session, surface wireless EMG sensors (Ultium Wireless EMG System, Noraxon, Inc., Scottsdale, AZ, USA) were fixed on the muscle belly of the VL, VM, and RF muscles of the dominant leg after skin preparation (shaving, abrading, and cleaning with alcohol). EMG signals were amplified (× 1.000), high-pass and low-pass filtered (10 and 500 Hz, respectively), and were sampled up to 4000 Hz with the common mode rejection ratio of < −100dB. EMG signals were then registered and analyzed using MyoRESEARCH 3 software (Noraxon, Inc., Scottsdale, AZ, USA) and EMG amplitude analysis instruction. To do so, EMG signals were normalized to the MIVC. The mean value of the EMG amplitude of the VL, VM, and RF muscles during the entire TTE under hypoxia was recorded.

### Hypoxic exposure

The hypoxic condition (O_2_ = 13%; equivalent to an altitude of 3600 m) was induced using a hypoxic air generator equipped with a semipermeable filtration membrane (GO_2_ Altitude ERA II, Biomedtech, Melbourne, Australia). The generator constantly pumped the hypoxic air into two 120-L Douglas bags. Participants wore an inflatable air cushion mask with a non-rebreathing valve positioned and fixed comfortably using a rubber port full-face harness. The mask was connected to the Douglas bags. SpO_2_ was also recorded using a fingertip pulse oximeter.

### Prolonged cognitive effort

Participants performed a modified computerized version of CWST (100% incongruent) for 30 min under hypoxia. The incongruent CWST consisted of four-color words (yellow, blue, green, and red) in different font inks (yellow, blue, green, and red) that were randomly displayed on a computer screen until the participant entered an answer and were followed by a 1500 ms interval. There were four colored buttons on the keyboard. The participants were instructed to press the button corresponding to the ink color displayed on the screen. There was an exception to this rule, however, if the word appeared in red ink, then the correct answer was pressing the button corresponding to the color word itself^60^. It is noteworthy that performing this modified CWST for 30 min has been shown to induce a state of mental fatigue^57–59^, including in professional road cyclists^60^.

### Transcranial direct current electrical stimulation (tDCS)

**Figure 5:**
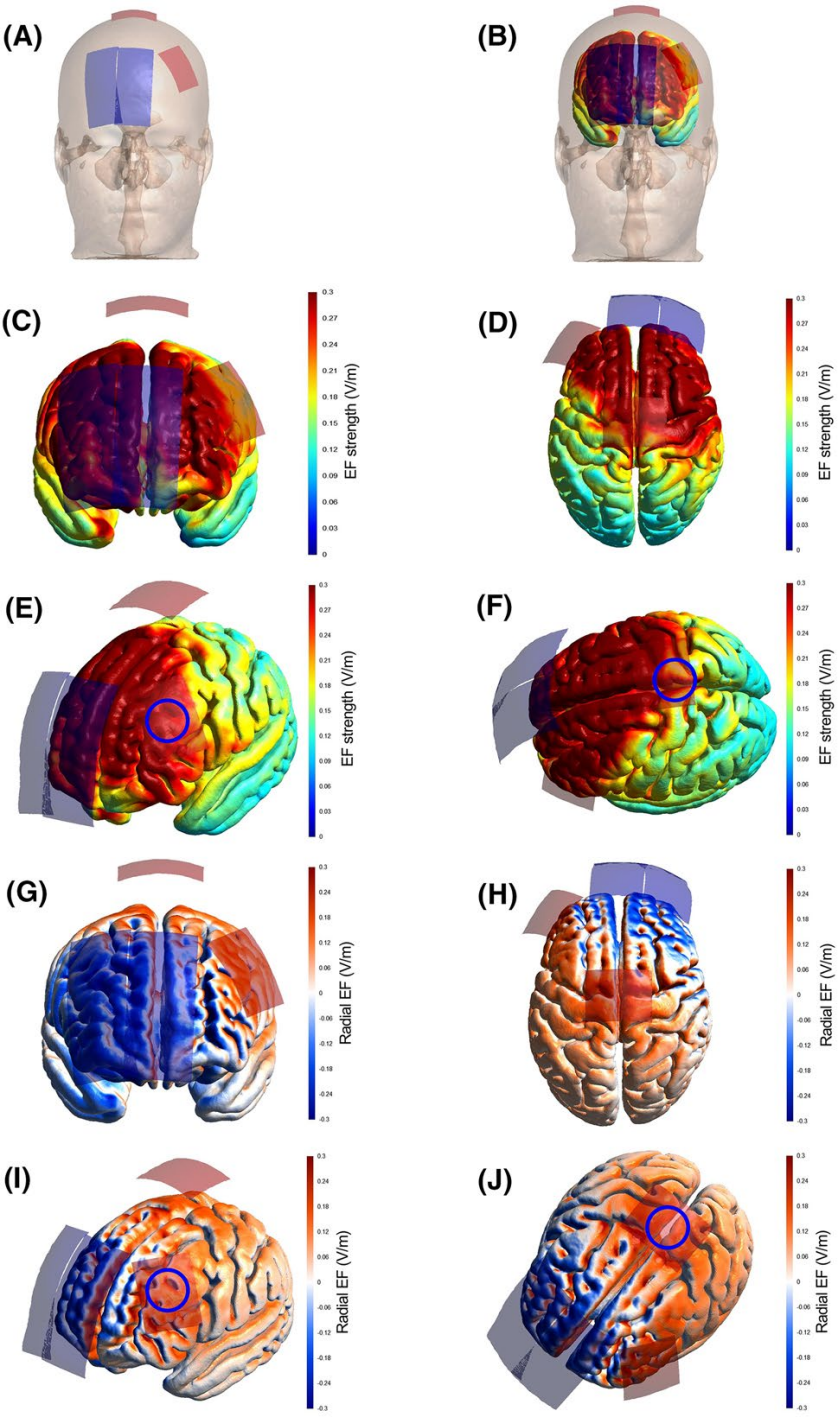
Strength and radial component of the electric field induced by tDCS. Finite Element Models derived from Magnetic Resonance Imaging in a head model (MNI152) of the strength and radial (normal to the cortical surface) component of the electric field (EF) induced by tDCS. Electrode montage targeting the simultaneous stimulation with anodal tDCS of the representation of the lower limbs in the primary motor cortex and the left dorsolateral prefrontal cortex (**A**, **B**), with red electrodes representing the anodes (5 × 4 cm) and blue electrodes representing the cathodes (9 × 4 cm). The EF strength is presented in the color-coded figures (**C**–**F**), with hotter colors indicating stronger EF and colder colors indicating the opposite. The radial EF is presented in the color-coded figures (**G**–**J**), where red color represents the electric current flowing into the cortex (i.e., inducing excitatory effects) and blue color represents the electric current flowing out of the cortex (i.e., inducing inhibitory effects). The study montage has reached the target areas with enough electric current strength to induce a neuromodulatory effect, as shown in figures (**E**) and (**F**) (blue circles roughly representing the target areas). Furthermore, the target areas were stimulated with the desired polarity (i.e., anodal current) to induce excitatory effects in the target regions, as shown in panels (**I**) and (**J**) (blue circles roughly representing the target areas).

Two-channel battery-driven stimulators (NeuroStim 2, Medina Tebgostar, Tehran, Iran) were used to apply tDCS over the target brain areas during the 3rd to 6th experimental sessions (two devices were used). Four carbon electrodes covered by saline-soaked surface sponges (NaCl 140 mmol dissolved in Milli-Q water) were used as anodes (5 × 4; 20 cm^2^) and cathodes (9 × 4; 36 cm^2^). The larger cathode electrodes were chosen to decrease the neuromodulatory effects of these electrodes. A 64-channel EEG cap with the international 10–20 EEG system positions was used to locate target areas over the scalp. The Unihemispheric Concurrent Dual-Site anodal tDCS** (**a-tDCS_UHCDS_) montage was used for simultaneously targeting M1 and DLPFC areas^55,56^. tDCS was applied offline (i.e., after the modified Stroop task) with 2 mA at each target for 20 min. One anode electrode was placed symmetrically over the Cz (2.5 cm on each side of the M1) targeting the representation of the lower limbs in M1 and the other anode electrode was placed vertically over F3 targeting the left DLPFC (Fig. 5A). The cathode electrodes were placed vertically over the supraorbital region, centered at AF4 and the other centered between Fpz and AFz (Fig. 5A). This montage was chosen based on recent studies showing that a-tDCS_UHCDS_ is an effective strategy for simultaneous stimulation of M1 and DLPFC yielding higher and long-lasting corticospinal excitability compared to the isolated stimulation of M1 and DLPFC^54,56^.

Two channels of the tDCS device were used for the simultaneous stimulation of these two regions. For anodal stimulation, the current was gradually ramped up for 30 s, maintained at 2 mA for 20 min, and then progressively ramped down for 30 s. In the sham condition (sh-tDCS), the same electrode position was applied, and the current was ramped up for 30 s, but the 2-mA current was only maintained for 30 s, and then ramped down for 30 s remaining off the rest of the time. This protocol has been shown to be adequate for blinding participants in tDCS studies^90–92^.

### tDCS modeling

The brain current flow during tDCS was calculated using a finite element model (FEM) following the standard pipeline in SimNIBS 4.0.0^93^. The magnetic resonance imaging (MRI) head model from the Montreal Neurologic Institute (MNI 152) available in the software was used for the simulation. MRI data were segmented into surfaces corresponding to the white matter (WM), gray matter (GM), cerebrospinal fluid (CSF), skull, and skin. The electrical conductivities of each segment were determined according to values previously established as follows: WM = 0.126 Siemens/meter (S/m), GM = 0.275 S/m, CSF = 1.654 S/m, bone = 0.010 S/m, and skin/scalp = 0.465 S/m^94^, rubber electrode = 29.4 S/m, and saline-soaked sponges = 1.000 S/m^94^. All information concerning the respective tDCS montages was entered into the software: current intensity = 2 mA for each anode; electrode position (+ F3 and + Cz/−Fpz and −AF4); electrode and sponge sizes (anodes 5 × 4 cm and cathodes 9 × 4 cm); electrode thickness = 1 mm; sponge thickness = 5 mm. The results of the simulations are presented in Fig. 5, in terms of the electric field strength (Fig. 5C–F) and radial (Fig. 5G–J) electric field (normal to the cortical surface), both of which are most important for neuromodulatory effects of tDCS^95^. As can be seen in Fig. 5C–F, the study montage has reached our target areas with enough electric current strength to induce a neuromodulatory effect (> 0.2–0.25 V/m)^96^. Furthermore, the target areas were stimulated with the desired polarity (i.e., anodal current) to induce excitatory effects in the target regions (Fig. 5G–J). Other areas such as the supplementary motor area, premotor cortex, and other prefrontal cortex were also stimulated in the current path from the anodal to the cathodal electrode. This spread electric current is a characteristic of tDCS applied with large rectangular pads (the so-called ‘conventional’ tDCS)^43^.

### tDCS-induced sensations and blinding assessment

Participants completed a questionnaire provided by Fertonani et al.^97^ after each session, listing the sensations and level of intensity experienced during the stimulation to measure tDCS-induced sensations, similar to previous studies^43,92,98^. Itching, pain, burning, warmth/heat, pitching, metallic/iron taste, fatigue, and other sensations (open question) were all listed on the questionnaire. The degrees were none (zero), mild (one), moderate (two), considerable (three), and strong (four). Participants also indicated whether these sensations affected their ability to perform the exercise (0 = not at all; 1 = slightly; 2 = considerably; 3 = much; 4 = very much); when the discomfort started (1 = beginning; 2 = at about the middle; 3 = towards the end); and when it stopped (1 = stopped quickly; 2 = stopped in the middle; 3 = stopped at the end). An aggregate variable (referred to as "discomfort" generated by tDCS) was computed as the total of the strength scores recorded for all sensations so that the discomfort variable ranged from 0 (lack of discomfort) to 28 (maximum discomfort). According to recent literature, the end of the study corrects guess rates, which indicates the percentage of participants that successfully guessed their experimental condition, which might lead to a misleading interpretation of blinding effectiveness^99,100^. It has been suggested to report the “active stimulation guess rate”, which indicates the percentage of participants who guessed they received the active treatment^100^. Hence, despite we report both correct and active stimulation guess rates, we will consider the latter as the measure of blind effectiveness^100^.

### Whole-body exhausting endurance task

Within the 3rd to 6th sessions, after 30 min performing CWST, followed by 20 min of tDCS, participants performed the TTE on a cycle ergometer (Cyclus 2, RBM Elektronik-Automation GmbH, Leipzig, Germany), all under hypoxia. Before TTE, participants warmed up cycling for 5 min at 45% PPO. After warming up, TTE started with a load of 60% of PPO and a 60 rpm cadence until exhaustion. The time between the beginning and interruption of the test was considered the TTE. HR and SpO_2_ were measured every minute during the task, and psychophysiological responses (i.e., RPE, FS, FAS) were reported every three minutes and at exhaustion. TTE was considered maximum if two of the following criteria were achieved: (1) ≥ 90% of maximal predicted HR (220-age), (2) inability to maintain the pedal cadence of ≥ 60 rpm for > 5 s, (3) RPE ≥ 90.

#### HR and SpO_2_

During the whole experimental session, HR was continuously monitored by the use of a chest strap (M430, Polar, Finland) connected to the cycle ergometer. SpO_2_ was also constantly measured and recorded by a fingertip pulse oximeter (Nonin Onyx II 9550, Nonin Medical, Plymouth, MN, USA).

### Ratings of perceived exertion

The 0–100 Borg scale was used to measure RPE. Participants received the instruction to report their RPE at the end of each stage and the point of exhaustion in the incremental test, and every 3 min and upon reaching the point of exhaustion in the TTE in hypoxia^101^. All these responses were used to calculate the average RPE in each experimental condition and used for statistical analyses.

### Affective responses and felt arousal

The affective responses were reported using the Feeling Scale (FS), which is a bipolar scale comprising eleven items ranging from  − 5 (very bad) to + 5 (very good), validated by Hardy and Rejeski^15^. The positive numbers represent pleasure, the negative numbers represent displeasure, and the zero represents a neutral affective valence. To measure the arousal, the Felt Arousal Scale (FAS) consisting of 6 items scored on a continuum from 1 (low arousal) to 6 (high arousal) was used^102,103^. Participants reported their affective responses and FAS every 3 min during the task and at exhaustion. All these responses were used to calculate average responses for these measures in each experimental condition and used for statistical analyses.

### Statistical analyses

The normal distribution of each data set was evaluated by the Shapiro–Wilk normality test. Values are presented as means and standard deviation (SD) or median and interquartile range (IQR) as stated. The Friedman test was used to compare tDCS-induced sensations, end-of-study guess rate accuracy, and active stimulation guess rate, followed by Wilcoxon signed-rank tests with a Bonferroni correction for pair-wise comparisons (0.05/6 = Bonferroni corrected *p* = 0.008), in case of significant differences (for tDCS-induced sensations, only).

One-way repeated measures ANOVA was performed to analyze the mean value of TTE, HR, SpO_2_, RPE, EMG amplitude, FS, and FAS during the TTE. Moreover, the mean value of CRT was analyzed using two-way repeated measures ANOVA (4 × 3 factorial design, 4 experimental conditions, and 3 time points). One-way repeated measures ANOVA was used for analyzing the simple main effect of condition and time when required. In case of a significant result, the post hoc test using Bonferroni correction for multiple comparisons was used for the pairwise comparisons. If the assumption of sphericity was violated, the Greenhouse–Geisser epsilon correction was applied. Partial eta squared (*ɳ*^*2*^_*p*_) was used as a measure of the effect size for the ANOVAs and interpreted as small (0.01–0.059), medium (0.06–0.139), or large (≥ 0.14). Cohen’s d was used as a measure of the effect size comparing all experimental conditions of interest (a-tDCS + DC, a-tDCS + WC, and sh-tDCS + DC) to the “complete” placebo (sh-tDCS + WC), and it was interpreted as small (0.20–0.49), medium (0.50–0.79), or large (≥ 0.80). The SPSS software version 26 (SPSS Inc., Chicago, IL, USA) was used for statistical analysis. The significance level for all tests was defined as *p˂0.05*.

### Ethics approval and consent to participate

The study was approved by the Ethics Committee of Razi University Kermanshah, Iran (IRCT id: IRCT20220417054556N1, 2022-07-27). All participants provided written consent to participate in the study.

## Data Availability

The datasets used and/or analyzed during the current study are available from the corresponding author upon reasonable request.
